# Atlas of mildly and highly insoluble matrisome driving liver fibrosis

**DOI:** 10.3389/fphar.2024.1435359

**Published:** 2024-09-02

**Authors:** Wen Zhang, Ning Zhang, Wenyue Wu, Hong Li, Hong You, Wei Chen

**Affiliations:** ^1^ Liver Research Center, Beijing Friendship Hospital, Capital Medical University, Beijing, China; ^2^ State Key Lab of Digestive Health, Beijing Friendship Hospital, Capital Medical University, Beijing, China; ^3^ National Clinical Research Center of Digestive Diseases, Beijing Friendship Hospital, Capital Medical University, Beijing, China; ^4^ Experimental and Translational Research Center, Beijing Friendship Hospital, Capital Medical University, Beijing, China; ^5^ Beijing Clinical Research Institute, Beijing Friendship Hospital, Capital Medical University, Beijing, China; ^6^ Chinese Institutes for Medical Research (CIMR), Beijing, China

**Keywords:** liver fibrosis, stiffness, decellularization, matrisome, solubility

## Abstract

The excessive deposition and cross-linking of core matrisome components typically result in abnormal remodeling of the extracellular matrix (ECM), leading to increased liver stiffness and worsening liver fibrosis. Exploring the biochemical properties of the ECM scaffold can deepen our understanding of the pathological mechanisms driving liver fibrosis and potentially facilitate the identification of therapeutic targets. While traditional sodium dodecyl sulfate (SDS)-based liver decellularization followed by proteomics can uncover the matrisome components within the ECM scaffold, it lacks the ability to reveal physicochemical characteristics like solubility. In our present study, using adult mouse liver as an example, we introduced a novel two-step workflow that combines our previously enhanced SDS (ESDS) decellularization with the conventional SDS method, enabling the identification of matrisome members with mild and/or high solubilities. Through this approach, we visualized the atlas of the mildly and highly insoluble matrisome contents in the adult mouse liver, as well as the regulatory network of highly insoluble matrisome that largely governs liver stiffness. Given the strong correlation between increased matrisome insolubility and heightened ECM stiffness, we believe that this methodology holds promise for future research focused on liver stiffness.

## Introduction

Extracellular matrix (ECM) within the liver is a complex microenvironment that coordinates tissue patterning and the fate of surrounding cells by offering a physical scaffold. Pathologically, both acute and chronic insults have the potential to reprogram liver ECM composition. In murine models, exposure to carbon tetrachloride for 48 h or a 2-week 3,5-diethoxycarbonyl-1,4-dihydro-2,4,6-collidine diet can alter the levels of ∼4.6% of liver ECM proteins (Klaas et al., 2016). Similarly, acute lipopolysaccharide injury or a 6-week ethanol diet can dramatically increase the number of liver ECM proteins by ∼25% (Massey et al., 2017). Additionally, our recent transcriptomic analyses have unveiled distinct profiles of ECM protein-encoding genes in prefibrotic, fibrotic and malignant liver livers (Chen et al., 2023a; Chen et al., 2021). These studies underscore that alterations in liver ECM composition are intricately linked to the specific nature of liver injuries. The remodeling of liver ECM often establishes pathological niches that trigger abnormal mechanical and biochemical transitions, fostering processes like inflammation, fibrogenesis, and potentially carcinogenesis. Conversely, restoring excessive or uncontrolled liver ECM remodeling is crucial for fibrolysis and has been confirmed to be negatively associated with the incidence of life-threatening liver-related events (LREs) (Chen et al., 2023a; Chen et al., 2021). Therefore, gaining a deeper understanding of the nature and magnitude of changes in ECM scaffold during liver fibrogenesis and fibrolysis may create emerging therapeutic opportunities for liver fibrosis.

Over the past decade, Alexandra Naba and her colleagues have defined ECM proteins as matrisome, encompassing collagens, glycoproteins, proteoglycans, ECM-affiliated proteins, ECM regulators, and secreted factors (Shao et al., 2023). Proteomics based on decellularized ECM scaffolds and enzymatic digestion can comprehensively assess both the qualitative and quantitative alterations in matrisome proteins within tissues (Krasny et al., 2016). Nowadays, various methodologies for tissue decellularization, such as physical, chemical, or biological treatments, have been developed and refined (Krasny et al., 2016; Chen et al., 2023b). With these advancements, exploring the matrisome in healthy and fibrotic livers has become feasible, which has the potential to uncover pathological mechanisms or identify valuable biomarkers for diagnosis or prognosis from the perspective of ECM (Taha and Naba, 2019). However, current proteomics methodologies, including the widely used ionic detergent sodium dodecyl sulfate (SDS) approach (Baiocchini et al., 2016), cannot measure another critical aspect of ECM beyond its qualitative and quantitative characteristics-its stabilization. In liver fibrogenesis, the covalent cross-linking usually occurs among intra- and inter-molecular protein chains of ECM proteins, thereby stabilizing ECM, a process that crucially involves members of the LOX family (Chen et al., 2020; Zhang et al., 2024). On one hand, ECM stabilization confers resistance to proteolytic degradation (Liu et al., 2016), which could be the reason why liver fibrosis patients do not regress after etiological removal. On the other hand, ECM stabilization increases its stiffness which is an independent risk factor correlated with the onset of decompensated cirrhosis or hepatocellular carcinoma (HCC) (John et al., 2024). Therefore, decoding ECM stabilization or stiffness at the molecular level will be conducive to forecasting the long-term prognosis.

Currently, the serial extraction and quantification of ECM collagens based on their solubility represent common practices in assessing collagen cross-links and ECM stabilization (Liu et al., 2016). In essence, collagens with varying degrees of cross-linking exhibit different solubility characteristics: acetic acid dissolves non-cross-linked or immaturely cross-linked collagens, pepsin dissolves moderately cross-linked collagens, and insoluble collagens do not dissolve in either acetic acid or pepsin. While this method provides an overall view of ECM stabilization, it falls short in identifying and quantifying all matrisome members with diverse solubility gradients, limiting its clinical utility. Therefore, our current study tried to develop a two-step method, involving refined SDS decellularization and proteomics to characterize mildly and highly insoluble matrisome members comprehensively. The goal is to establish a technical framework for understanding ECM proteomic studies in liver fibrosis.

## Methods and materials

### Liver matrisome profiling

Adult male mice (C57BL/6J background, 10 weeks) were purchased from Beijing HFK Bioscience Co., LTD. (Beijing, China). Liver tissues were harvested following euthanasia by neck dislocation under anesthesia and stored at −80°C for SDS or enhanced SDS (ESDS) decellularization, as described previously (Chen et al., 2023b). The typical steps between the two methods included plasma removal, SDS decellularization, residual SDS removal, and washing. However, in the ESDS decellularization workflow, we introduced additional physical treatments, including a freeze-thawing cycle and needle puncturing prior to SDS treatment, increased the concentration of the SDS solution, added non-ionic detergents such as 0.1% Triton-X and Tween 20, and accelerated the shaking procedure. The decellularized ECM scaffolds were utilized for subsequent enzymatic digestion to produce peptide fragments, with key steps involving reduction, alkylation, deglycosylation, digestion, acidification, and desalting. Peptide samples underwent proteomics profiling using label-free liquid chromatography-tandem mass spectrometry (LC-MS/MS, Beijing Qinglian Biotech Co., Ltd., Beijing, China). SDS or ESDS decellularization-based matrisome profiling was repeated twice using livers from two adult male mice. The mouse studies were approved by the Ethics Committee of Beijing Friendship Hospital, Capital Medical University, and conducted in accordance with the ARRIVE guidelines.

### MS data preprocessing and bioinformatics analysis

The MS data was preprocessed using the Proteome Discoverer suite (version 2.4, Thermo Fisher Scientific) with the Sequest HT search engine for protein identification, as outlined previously (Chen et al., 2023b). Proteins identified through LC-MS/MS were searched against mouse matrisome lists defined by Alexandra Naba et al. (Shao et al., 2023). Matrisome proteins consistently identified in repeated experiments were selected for further analysis. VENNY 2.1 tool (https://bioinfogp.cnb.csic.es/tools/venny/index.html) was employed to perform Venn analysis of matrisome proteins shared between SDS or ESDS decellularization-based matrisome profiling. STRING database (https://cn.string-db.org/) was utilized to retrieve the potentially interactive relationships among the input matrisome members, visualized through Cytoscape v3.9.1 (https://cytoscape.org/). Enriched Kyoto Encyclopedia of Genes and Genomes pathways of the input matrisome members were analyzed using the DAVID web tool (https://david.ncifcrf.gov/), with pathways displaying a Benjamini-corrected *p*-value < 0.05 being visualized using the *ggplot2* R package. The clinical relevance of the corresponding encoding genes of the input matrisome members was assessed using the GSE15654 dataset (Hoshida et al., 2013), containing 216 cirrhotic liver samples retrieved from the Gene Expression Omnibus database (https://www.ncbi.nlm.nih.gov/geo/). Potential associations between the matrisome members of interest and the probability of decompensation occurrence, HCC onset, or death were analyzed using the *survival* R package. The log-rank test was used to determine the statistical significance, and a *p*-value < 0.05 was considered statistically significant.

## Results

**FIGURE 1:**
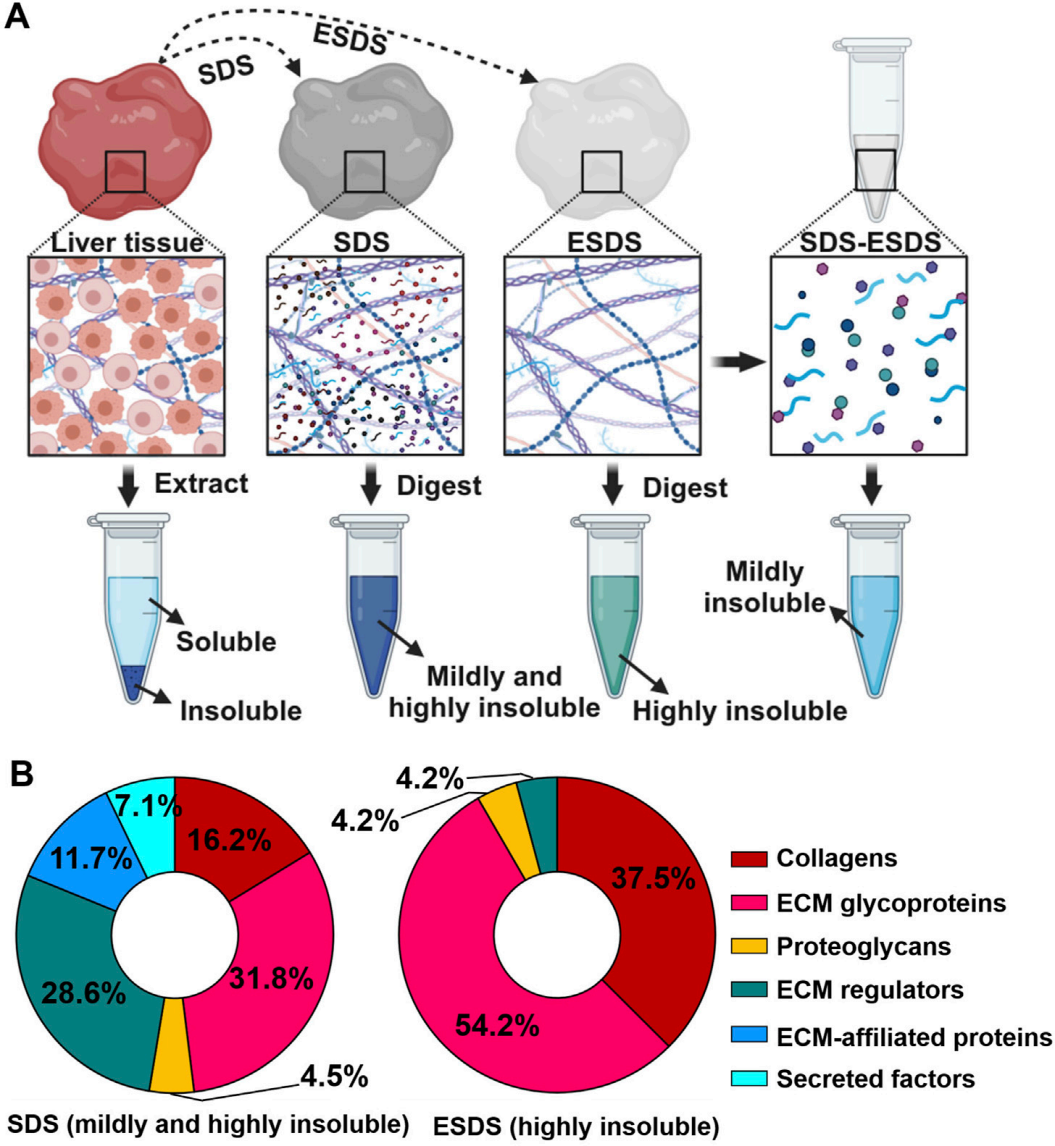
Identification of mildly and highly insoluble matrisome members. **(A)** Schematic diagram illustrating the coverage of mildly and highly insoluble matrisome components in adult mouse liver through the combination of the commonly used SDS pipeline and our ESDS pipeline. **(B)** The ratio of the number of members in each matrisome category to the total number of matrisome members identified by the commonly used SDS pipeline and our ESDS pipeline. Matrisome categories are color-coded.

The ESDS decellularization workflow-based proteomic profiling has proven effective in characterizing highly insoluble matrisome proteins within the liver ECM scaffold (Chen et al., 2023b). Moreover, the mildly insoluble matrisome proteins present in the liver ECM scaffold can also be identified through the integration of our ESDS decellularization and the commonly used SDS method. As depicted in Figure 1A, matrisome proteins exclusively identified through the SDS pipeline were categorized as mildly insoluble; conversely, those exclusively identified by ESDS pipeline were classified as highly insoluble. In contrast, all the ECM-associated proteins, secreted factors, and the majority of proteoglycans and ECM regulators were categorized as mildly insoluble matrisome components within the mouse liver ECM scaffold, as they were predominantly identified through the SDS pipeline rather than the ESDS pipeline. (Figure 1B).

**FIGURE 2:**
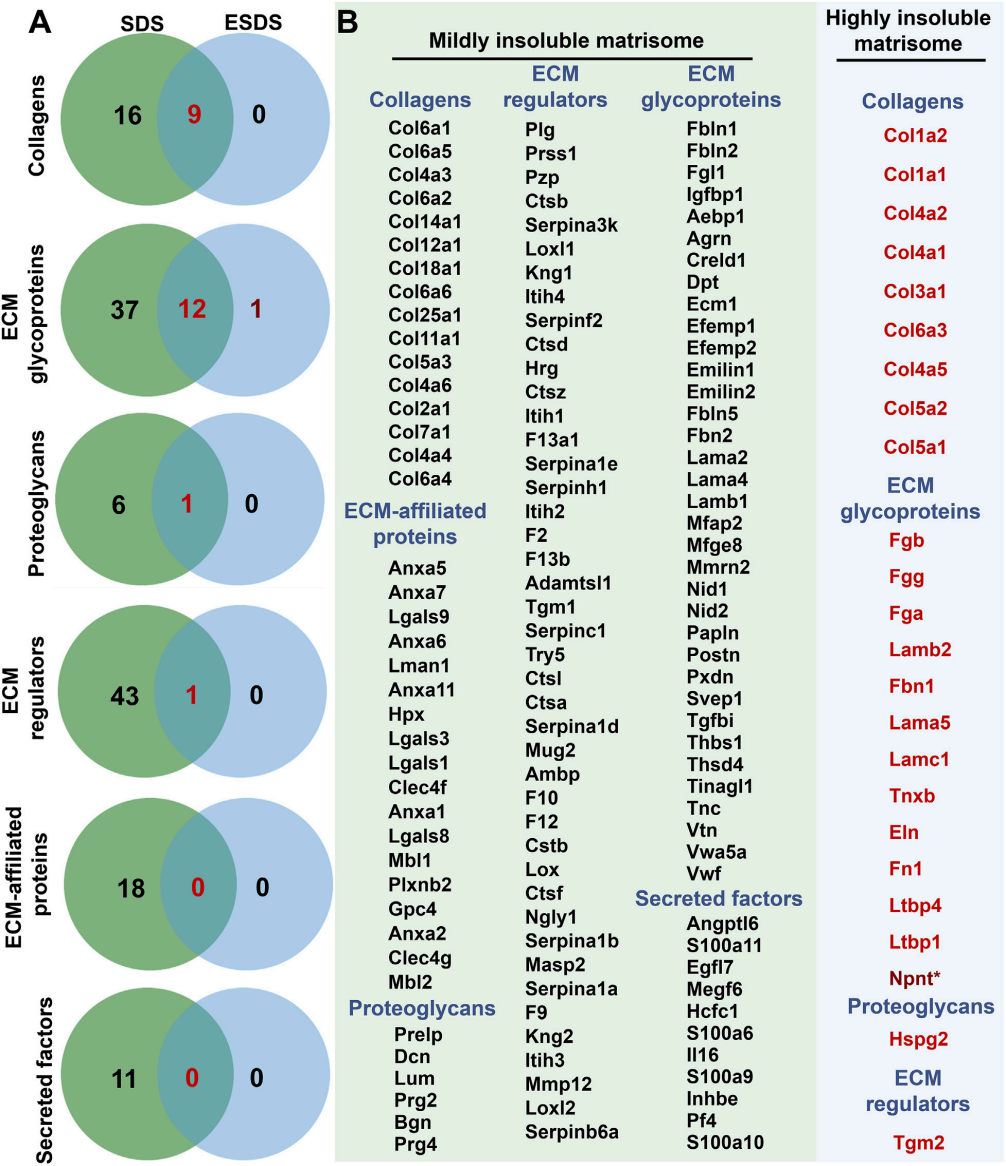
Mildly and highly insoluble matrisome members in the adult mouse liver ECM scaffold. **(A)** Venn analyses of the insoluble matrisome proteins in each category identified based on the SDS and ESDS decellularization workflows. **(B)** Detailed information on mildly and highly insoluble matrisome proteins in the adult mouse liver ECM scaffold. * represents proteins only detected using the ESDS pipeline.

Specifically, in adult mouse liver, a total of 16 collagens, 37 ECM glycoproteins, 6 proteoglycans, 43 ECM regulators, 18 ECM-affiliated proteins, and 11 secreted factors were identified as mildly insoluble matrisome components. Additionally, a subset of these components, including 9 collagens, 12 ECM glycoproteins, 1 proteoglycan, and 1 ECM regulator, was detected as both mildly and highly insoluble matrisome proteins (Figure 2A). Detailed information regarding these mildly and highly insoluble matrisome proteins in the adult mouse liver ECM scaffold is presented in Figure 2B.

**FIGURE 3:**
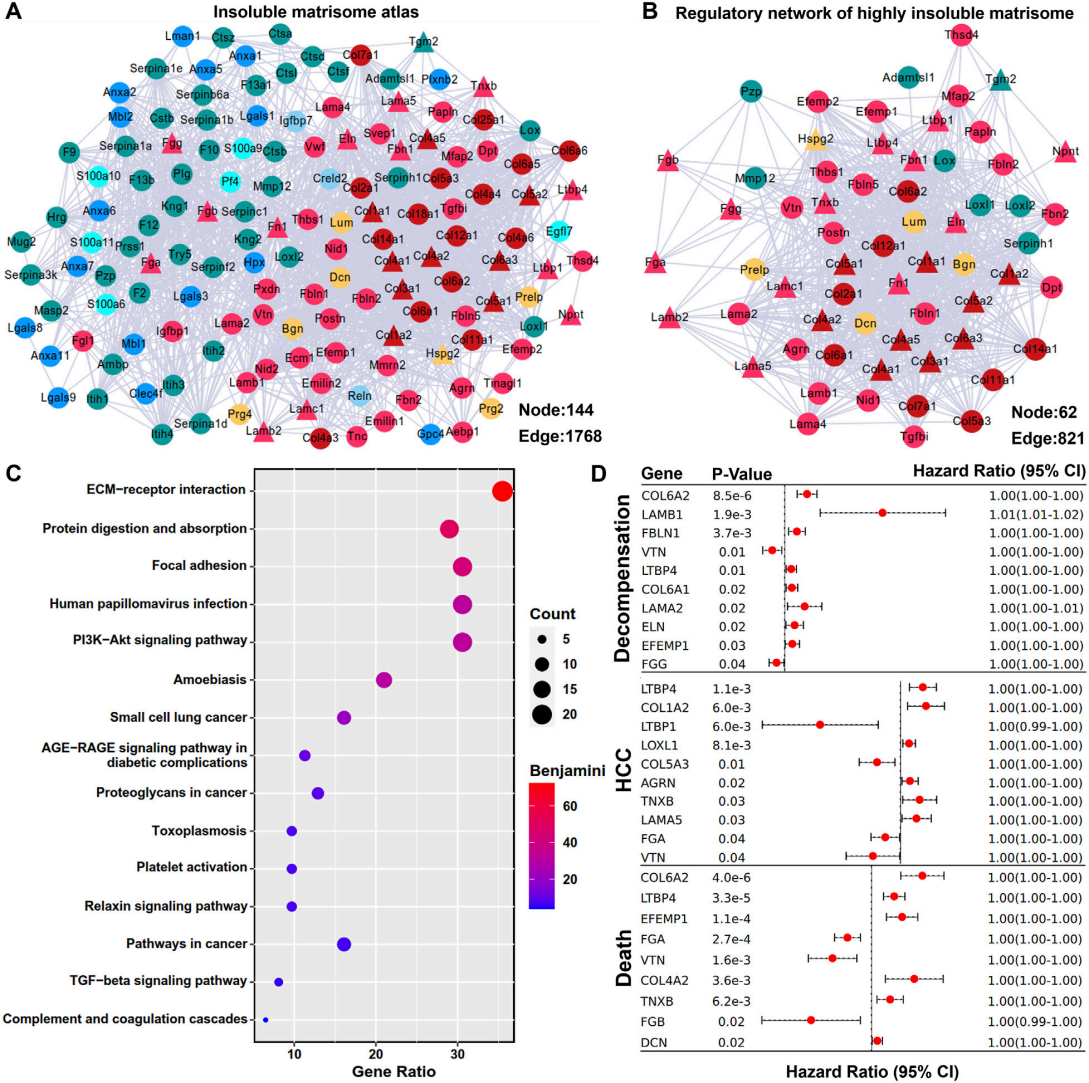
Regulatory network of highly insoluble matrisome members. **(A)** Interactive atlas of the insoluble matrisome in adult mouse liver ECM scaffold. The mildly insoluble matrisome members are marked using circular nodes and the highly insoluble matrisome members identified by ESDS workflow are marked using triangular nodes. Each matrisome category is color-coded as indicated in Figure 1B. Connections between nodes represent interactive relationships. **(B)** Regulatory network of highly insoluble matrisome members in adult mouse liver ECM scaffold. The first neighbors of the highly insoluble matrisome memberss (triangular nodes) are marked by circular nodes, with each category also differentiated by color coding. **(C)** Functional enrichment analysis of the regulatory network of highly insoluble matrisome members. Benjamini-corrected *p*-value < 0.05 was considered as statistically significant. **(D)** Prognostic evaluation of all the highly insoluble matrisome members in liver fibrosis. The log-rank test was employed to evaluate the prognostic significance of these members, with results deemed significant at a *p*-value < 0.05, which are displayed in forest plots. CI, confidence interval.

The interactive atlas of insoluble matrisome in adult mouse liver ECM scaffold was predicted and visualized using the STRING database and Cytoscape software. It was characterized as a dense regulatory network with 1768 potential interactions (Figure 3A), representing a complex ECM environment crucial for maintaining liver homeostasis. Within the insoluble matrisome atlas, the core matrisome members (collagens, ECM glycoproteins, and proteoglycans) were observed to be closely interconnected with each other (Figure 3A). The first interactive neighbors of the highly insoluble matrisome members were retrieved, followed by the construction of the regulatory network of the highly insoluble matrisome, comprising 62 matrisome members with 821 interactions (Figure 3B). Subsequent functional enrichment analysis revealed that the regulatory network of highly insoluble matrisome was associated with various fibrogenesis and carcinogenesis pathways, including ECM-receptor interaction, focal adhesion, PI3K-Akt signaling pathway, platelet activation, TGF-beta signaling pathway, complement and coagulation cascades, small cell lung cancer, proteoglycans in cancer, and pathways in cancer (Figure 3C). Additionally, the prognostic values of highly insoluble matrisome members were assessed in liver fibrosis patients using the transcriptomic expression matrix and follow-up clinical data from the GSE15654 dataset (Hoshida et al., 2013). As shown in Figure 3D, perturbation of several highly insoluble matrisome members was associated with the clinical prognosis of liver fibrosis, including the probabilities of decompensation occurrence, HCC onset or death.

## Conclusions

Pathological ECM remodeling typically results in the formation of a fibrous scaffold, which increases local tissue stiffness and subsequently influences the behaviors of surrounding cells through mechanical forces. Additionally, this fibrous ECM scaffold serves as a reservoir for signaling molecules embedded within the ECM, transmitting chemical signals through cell surface adhesion receptors (Hynes, 2009). Therefore, conducting ECM discovery proteomic analysis to explore the differences between normal and fibrotic livers is highly recommended, with the goal of identifying potential therapeutic targets or diagnostic biomarkers for liver fibrosis. Traditionally, ECM collagen fractions with varying solubilities have been roughly assessed using progressively harsher conditions, including neutral salt, acetic acid, and pepsin (Liu et al., 2016). With the rapid advancements in liver decellularization and proteomics, precise quantitative analysis of all extracellular matrisome members is now possible (Krasny et al., 2016). However, the solubility properties of these members have often been overlooked. Given that covalent cross-linking occurs physiologically and pathologically, the solubility properties of matrisome members vary. Consequently, our current study has developed a two-step workflow integrating our ESDS decellularization with the commonly used SDS method, enabling the identification of matrisome members with mild and/or high solubility. As the increased concentration of SDS aids in solubilizing mildly insoluble ECM proteins (Naba et al., 2015), in our ESDS decellularization method, we have enhanced the SDS concentration from 1% to 1.5% (Chen et al., 2023b). Additionally, it is advisable to perform needle puncture and freeze-thaw cycles before initiating the decellularization process. This step is crucial to facilitate deep tissue penetration by the detergent solution, ensuring optimal contact with the decellularization reagent. The subsequent proteomic analysis has demonstrated that ESDS decellularization not only enhances the efficiency of cell removal but also facilitates the extraction of mildly insoluble ECM proteins (Chen et al., 2023b).

In the present study, we tested the newly proposed two-step workflow using adult mouse liver as an example. For the first time, we revealed the atlas of mildly and highly insoluble matrisome in the liver of mice with a C57BL/6J background that has been extensively used as a tool for studying liver fibrosis. As anticipated, the majority of highly insoluble matrisome constituents within the ECM scaffold of adult mouse liver are core members, including collagens (Col1a1, Col1a2, Col3a1, Col4a1, Col4a2, Col4a5, Col5a1, Col5a2 and Col6a3), laminins (Lama5, Lamb2 and Lamc1), fibrinogens (Fga, Fgb and Fgg), elastin (Eln), fibronectin (Fn1), and fibrillin (Fbn1). Excessive deposition and cross-linking of these core members usually lead to increased ECM stiffness (Ortiz et al., 2021; Chen et al., 2019). To identify the key matrisome components governing liver stiffness, we constructed a regulatory network of highly insoluble matrisome by identifying their primary interactive neighbors (mildly insoluble) from the total matrisome atlas. Notably, within this network, lysyl oxidases (Lox, Loxl1, Loxl2) and transglutaminases (Tgm2) are well-known enzymes for the covalent cross-linking of ECM structural proteins (Chen et al., 2020); besides, ECM glycoproteins such as fibulins (Fbln1, Fbln2 and Fbln5) and EGF containing fibulin extracellular matrix proteins (Efemp1and Efemp2) have been confirmed to be essential for the assembly of elastic fibers that highly contribute to increased ECM stiffness (Karamanos et al., 2021). Therefore, focusing on the regulatory network of highly insoluble matrisome may contribute to decoding ECM stiffness-sensitive molecules, warranting further investigation.

Clinically, the increased liver stiffness, due to the excessive deposition of highly insoluble matrisome, is associated with the risk of decompensation, liver cancer, and death in patients with liver fibrosis (John et al., 2024). Moreover, liver stiffness is increasingly acknowledged as a crucial factor in HCC progression and the effectiveness of immunotherapy (Mai et al., 2024). Thus, a comprehensive analysis of mildly and highly insoluble matrisome components in fibrotic or malignant livers not only unveils the predominant insoluble matrisome contributing to ECM stiffening, but also sheds light on mildly insoluble matrisome members that aid in covalent cross-linking or ECM stabilization. This insight not only offers potential biomarkers for predicting outcomes but also identifies therapeutic targets for reducing liver stiffness, thereby lowering the incidence of liver-related complications or boosting cancer immunotherapy. In addition, our recent study has demonstrated that histologic fibrosis regression is associated with decreased LREs in liver fibrosis patients with chronic hepatitis B virus infection after antiviral therapy (Sun et al., 2024). However, the histologic evaluation by pathologists is somewhat subjective, susceptible to biopsy size, sampling disparity and error, and inter- and intra-observer variation (Chen et al., 2023a). Our newly proposed two-step method offers a precise approach to evaluate histologic regression by analyzing mildly and highly insoluble matrisome members. Importantly, this method holds promise for clinical drug evaluation as it can detect subtle changes in fibrotic livers beyond traditional pathological assessments, by decoding both mildly and highly categories of insoluble matrisome components.

Honestly, our proposed two-step workflow for characterizing mildly or highly insoluble matrisome members has certain limitations that need further attention. Firstly, identifying mildly insoluble matrisome members may require two rounds of mass spectrometry, which can be costly. Exploring the feasibility of labeling peptides from the SDS and ESDS workflows with different isobaric tags, mixing them, and analyzing them in a single mass spectrometry experiment warrants additional investigation. Secondly, while the ESDS workflow can achieve the highest ECM purity, the SDS workflow may struggle to prevent cellular debris contamination. Therefore, refining the SDS workflow to enhance ECM purity is crucial to avoid overestimating the abundance of mildly insoluble matrisome members. Despite these challenges, the two-step workflow can delineate matrisome characteristics in terms of content and solubility. We believe it holds promise for future studies focused on ECM stabilization and stiffness.

## Data Availability

The datasets presented in this study can be found in online repositories. The names of the repository/repositories and accession number(s) can be found below: https://doi.org/10.6084/m9.figshare.25858159.v1.
